# Home-based upper limb stroke rehabilitation mechatronics: challenges and opportunities

**DOI:** 10.1186/s12938-023-01133-8

**Published:** 2023-07-09

**Authors:** Shane Forbrigger, Vincent G. DePaul, T. Claire Davies, Evelyn Morin, Keyvan Hashtrudi-Zaad

**Affiliations:** 1 Department of Electrical and Computer Engineering, Queen’s University, Kingston, Canada; 2 School of Rehabilitation Therapy, Queen’s University, Kingston, Canada; 3 Department of Mechanical and Materials Engineering, Queen’s University, Kingston, Canada

**Keywords:** Rehabilitation robotics, Physical patient–robot interaction, Design methods

## Abstract

Interest in home-based stroke rehabilitation mechatronics, which includes both robots and sensor mechanisms, has increased over the past 12 years. The COVID-19 pandemic has exacerbated the existing lack of access to rehabilitation for stroke survivors post-discharge. Home-based stroke rehabilitation devices could improve access to rehabilitation for stroke survivors, but the home environment presents unique challenges compared to clinics. The present study undertakes a scoping review of designs for at-home upper limb stroke rehabilitation mechatronic devices to identify important design principles and areas for improvement. Online databases were used to identify papers published 2010–2021 describing novel rehabilitation device designs, from which 59 publications were selected describing 38 unique designs. The devices were categorized and listed according to their target anatomy, possible therapy tasks, structure, and features. Twenty-two devices targeted proximal (shoulder and elbow) anatomy, 13 targeted distal (wrist and hand) anatomy, and three targeted the whole arm and hand. Devices with a greater number of actuators in the design were more expensive, with a small number of devices using a mix of actuated and unactuated degrees of freedom to target more complex anatomy while reducing the cost. Twenty-six of the device designs did not specify their target users’ function or impairment, nor did they specify a target therapy activity, task, or exercise. Twenty-three of the devices were capable of reaching tasks, 6 of which included grasping capabilities. Compliant structures were the most common approach of including safety features in the design. Only three devices were designed to detect compensation, or undesirable posture, during therapy activities. Six of the 38 device designs mention consulting stakeholders during the design process, only two of which consulted patients specifically. Without stakeholder involvement, these designs risk being disconnected from user needs and rehabilitation best practices. Devices that combine actuated and unactuated degrees of freedom allow a greater variety and complexity of tasks while not significantly increasing their cost. Future home-based upper limb stroke rehabilitation mechatronic designs should provide information on patient posture during task execution, design with specific patient capabilities and needs in mind, and clearly link the features of the design to users’ needs.

**Supplementary Information:**

The online version contains supplementary material available at 10.1186/s12938-023-01133-8.

## Background

Stroke is the third most common cause of disability in the world, with 25.7 million stroke survivors worldwide [1]. The total number of stroke survivors is expected to increase with a demographic shift to an older population, and reduced stroke mortality due to improved health care and public health initiatives [2, 3]. The increasing number of individuals living with stroke poses a challenge to healthcare systems. Among the many potential impairments experienced by stroke survivors, loss of upper limb motor function is the most prevalent, affecting 77.4% of stroke survivors [4]. Upper limb impairment persists for longer than 6 months for 89% of stroke survivors who lose upper limb function [5], having a significant effect on their quality of life [6]. Stroke survivors can recover function through the process of neuroplasticity [7], which can be accelerated through repetitive practice in rehabilitation therapy [8, 9]. As a result, more people than ever need access to rehabilitation services.

Access to rehabilitation services is more difficult for people who live far from clinics, in rural or under-resourced areas. Additionally, the recent COVID-19 pandemic has reduced access further: policies to reduce transmission through physical and social distancing make rehabilitation activities, often requiring close contact between patients and therapists, difficult or even impossible for outpatients. The healthcare system must adapt to this challenge; and one promising avenue is the use of rehabilitation mechatronics, such as robot-assisted rehabilitation therapy, in the home environment. Mechatronic devices in this work refers to mechanisms with which a patient can physically interact that incorporate sensors in their design and may also include actuators. Robots are a particular subset of mechatronic device: a mechanism with sensors and actuators joined by a control loop.

### A brief history of upper limb rehabilitation mechatronics

**Fig. 1:**
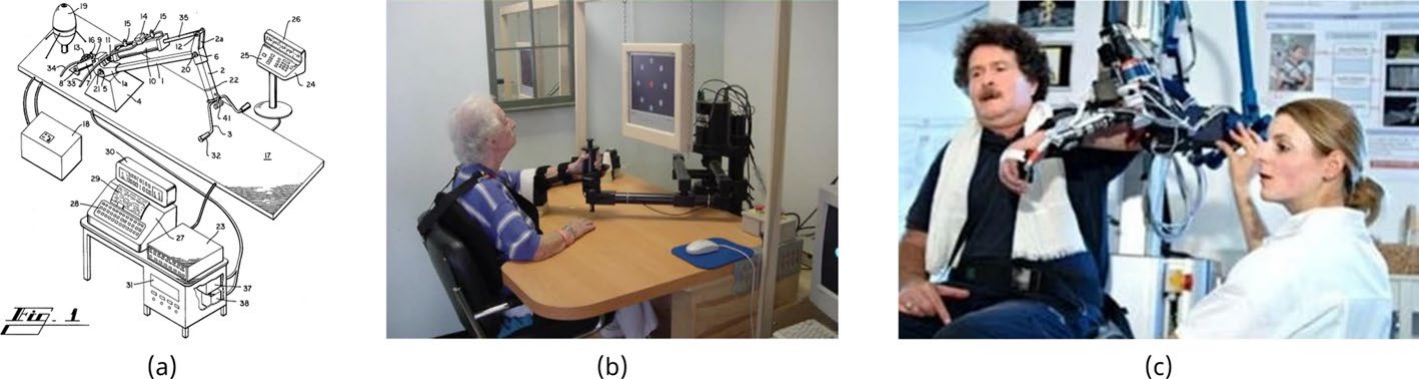
Early in-clinic robotic rehabilitation devices: **a** a robotic exercise machine [10], Public Domain; **b** *MIT-MANUS* [11], licensed under CC-BY$-$2.0; **c** *ARMin* [12], licensed under CC-BY$-$2.0

Mechatronic rehabilitation devices have been in development since the late 1970s. One of the earliest examples was a large, hydraulically powered arm which required an entire room and a team of therapists and technicians to operate [10, 13]. In the early 1990s the *MIT-MANUS* was introduced [14]. The *MIT-MANUS*, often cited as the seminal rehabilitation robot, occupied a desk-sized workstation and allowed a patient to participate in planar arm exercises under the supervision of a single therapist or technician. Even in the early days of development, the patent for the device suggested future modules for the wrist and hand, and the home environment being the eventual goal [15]. The *MIT-MANUS* was later commercialized as the *InMotion ARM* (www.bioniklabs.com), with the additional modules becoming the *InMotion ARM/HAND*. Contrary to the aspirations of the patent, the *InMotion ARM* has remained a clinic-based device. Over time more complex rehabilitation robots were developed. In the mid-2000s, the *ARMin* robot [16, 17] was one of the first joint-based upper limb rehabilitation devices (often referred to as an exoskeleton). Images of these early rehabilitation devices are shown in Fig. 1. A later version of the ARMin added control of the shoulder joint [18], and was commercialized as the *ArmeoPower* (www.hocoma.com), a clinic-based device.

Starting with the development of the *MIT-MANUS*, the effectiveness of robotic rehabilitation for stroke has been of significant research interest. The initial study of the *MIT-MANUS* [19] showed promise that robotic rehabilitation could be an effective stroke rehabilitation treatment. Since then, a large number of studies have found that dose-matched robotic stroke rehabilitation promotes upper limb recovery similar to traditional therapy [20–22] and kinematic data from robotic rehabilitation devices can be used to assess upper limb function [23–25]. Robot-assisted rehabilitation therapy has been recommended for treatment of upper limb paresis by the American Heart Association since 2016 [26].

While clinic-based rehabilitation robots are becoming more commonplace, home-based rehabilitation robots are not as widespread. A survey of upper limb rehabilitation robots in 2014 predicted that home-based devices would become more prevalent as demand for such devices increased [27]. Accordingly, the past 12 years have seen some of the first user studies of rehabilitation robots in the home environment [28] and some of the first commercialized devices advertised for home use such as the *ArmAssist* (www.armassist.eu). Compared to the clinic-based devices, home-based devices face unique challenges in their development and adoption. The most significant challenges are: safety, cost, space requirements, and independent ease-of-use [28].

### The need for home-based rehabilitation mechatronics

Currently, the majority of stroke rehabilitation is provided in hospital settings. The intensity of therapy decreases significantly as individuals with stroke are discharged to the community [29]. In Canada, robot-assisted rehabilitation therapy is particularly promising in settings where stroke survivors are currently not receiving enough conventional therapy, such as in the home [29]. Many patients need therapy even after being discharged [5, 30], thus providing home-based rehabilitation to stroke survivors offers several advantages.

Providing home-based rehabilitation to stroke survivors offers many advantages. For the stroke survivors themselves, the home environment is comfortable, familiar, and closer to family and friends. For the healthcare system, delivering therapy in stroke survivors’ homes results in a lower likelihood of patient readmission [31]. Delivering therapy in stroke survivors’ homes also removes barriers to access for those who have difficulty travelling.

The recent COVID-19 pandemic has demonstrated another advantage of home-based rehabilitation mechatronics: they allow therapy to continue for patients when physical distancing measures are in place. The majority of stroke survivors are older adults with a variety of underlying health conditions [29], which makes them a vulnerable population for COVID-19 and other life-threatening communicable diseases [32]. Home-based rehabilitation robots would allow stroke survivors to perform therapies alone or with a household member that would normally require the presence of a human therapist, enabling them to continue their therapy while still self-isolating. Therefore, the development and adoption of home-based rehabilitation robots are critical to maintaining and improving rehabilitation outcomes for patients while simultaneously protecting them from COVID-19 and future pandemics, as well as other potentially serious infections.

While there are many advantages to providing home-based therapy, there are challenges to implementation. Home-based programs need frequent, high-repetition activities to be most effective: at least 45 min per day, 2–5 days per week [33]. Home-based therapy, where a therapist must travel between patients’ homes, is less time-efficient for the therapist. Additionally, goal-directed, task-based practice, where therapy activities include elements of functional tasks, are most effective for recovery [34] but require more instruction than exercise-based therapy [35]. Home-based rehabilitation mechatronics can help mitigate these limitations: automated therapy sessions can increase the number of therapy repetitions without needing to increase the number of in-person visits, and provide immersive, goal-oriented activities for patients to practise.

**Fig. 2:**
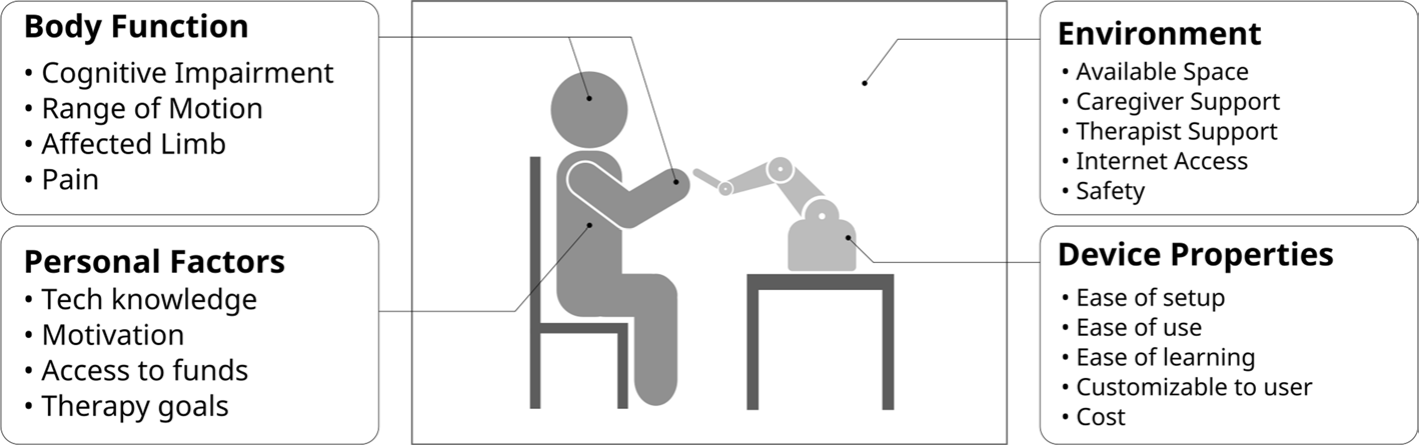
Examples of contextual factors in the design of an at-home rehabilitation robot, synthesized from [36–38]. The Device Properties interact with the contextual factors to determine the user’s experience. While these factors also apply to in-clinic devices, the Personal and Environment factors are more impactful in the home setting compared to a controlled clinic

The requirements for the design of home-based rehabilitation mechatronic devices have been the subject of some research. Sivan et al. [36, 37] found that the World Health Organization’s International Classification of Functioning, Disability, and Health (ICF) [39] provides a useful framework for identifying user needs in the home environment. The ICF is based on a biopsychosocial model of disability, where a person’s function is affected by the interaction between their health condition, impairments, and contextual factors, which include environmental and personal factors [39]. A variety of contextual factors which impact a stroke survivor’s use of rehabilitation technologies have been identified through interviews with stroke survivors and therapists [36–38]. Some of these factors are summarized in Fig. 2.

The design of a home-based rehabilitation mechatronic device must relate the properties of the device to the contextual factors within which it will be situated. An important distinction between home-based and in-clinic devices is that the personal and environment factors are better managed in the clinic: the environment itself is controlled, trained staff are available to assist the patient, and the patient is directly instructed in the activity. Therefore, while in-clinic devices can focus largely on a patient’s body function and anatomy in their design, home-based devices must weigh the personal and environmental factors more heavily in their design process. Consider how the Device Property “ease of setup” differs between a clinic and a home environment. In a clinic, a trained therapist or technician can set up or configure the device, therefore the bar for what is ‘easy’ differs from the home environment where the stroke survivor may have to set up the device themselves with an impaired arm, or other issues.

### Objective

The objective of this review is to investigate the capabilities of upper limb rehabilitation mechatronic devices designed for home use that have been developed in the past 12 years and identify the remaining challenges to further development. The reason for considering mechatronic devices more broadly rather than robots specifically is that not all stroke patients require the added assistance or resistance of an actuated device. Therefore, creative solutions that balance the cost of adding additional features versus the specific needs of the end-user are worth considering in the review.

While in-clinic devices have demonstrated that robot rehabilitation can be an efficacious treatment, the effectiveness of a treatment depends on its ability to meet user needs in a given environment [40]. Home-based rehabilitation devices have greater restrictions on their design than in-clinic devices, specifically in cost, safety, ease-of-use, and space requirements. Since home-based devices are used by a single stroke survivor over a prolonged period of time, costs and cost-efficiency must be considered more than in a clinic setting, where devices may be used by more than one patient over a single day. They also must be safer and easier to use than in-clinic devices since there are no therapists or technicians available to assist in their operation. Finally, they must be sufficiently light, portable, and compact to be easily installed in a stroke survivor’s home. These properties distinguish home-based devices from clinic-based devices, therefore they should be considered separately.

Previous reviews have either considered rehabilitation devices intended for both in-clinic and at-home use [27, 41], or only considered devices that have undergone in-home user studies [28]. As already stated, since home-based rehabilitation devices face unique constraints on their design, they should be considered separately from in-clinic devices. Additionally, the focus of this review is on the function of devices rather than an analysis of their performance. Therefore, this review will consider all devices that have been developed in the past 12 years, regardless of whether they have had user testing, in order to identify the latest developments in this area.

The contributions of this article are as follows. First, it summarizes the state of the art in home-based upper limb stroke rehabilitation mechatronic devices reported on over the past 12 years. Second, it compares existing devices based on their capabilities relative to stakeholder needs. Finally, it summarizes the limitations of existing devices and provides recommendations for the development of future devices reflected by user needs.

## Methods

The scope of the review was limited to mechatronic devices, mechanisms with which a patient can physically interact that incorporate sensors in their design and may also include actuators, collectively referred to as ‘devices’, specifically designed to provide exercise therapies for upper limb rehabilitation in a stroke survivor’s home. The devices must be purpose-built to facilitate therapeutic activities, not an assistive device that aids a person in their activities of daily life (ADL), nor an assessment system that solely measures a person’s function. Rehabilitation games created on existing platforms such as video game systems, a camera system, or cell phones were excluded. Papers describing incomplete designs, such as the design of an actuator for an unrealized future device, were also excluded.

**Fig. 3:**
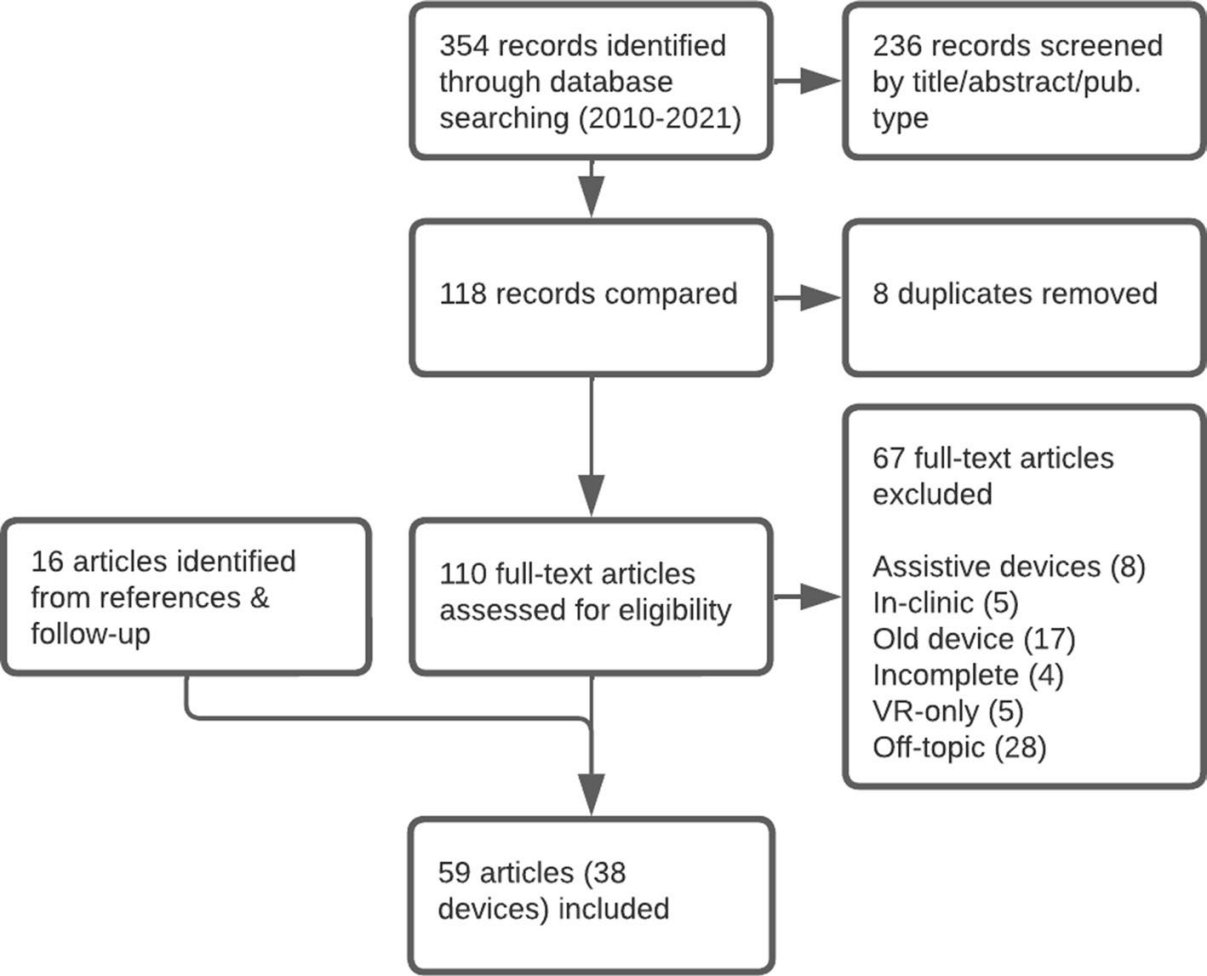
The flow diagram of the study

Three databases were searched: IEEEXplore, ScienceDirect, and PubMed. Searches were performed using a combination of the following keywords: “upper extremity”, arm, “upper limb”, stroke, rehabilitation, therapy, training, robot (and variations such as robotic) and home. Records were limited to the period between January 1, 2010 and December 3, 2021, when the final search occurred. This time period was chosen because the field of rehabilitation mechatronics is developing rapidly, therefore a 12-year period was considered sufficient to capture the state of the art. Papers were first filtered by title and abstract, then further filtered by full-text content. The flow diagram of the study is shown in Fig. 3. The search and filtering process was conducted by author S.F. The final list includes both research and commercial devices. To the best of the authors’ knowledge, the *Haptic Theradrive*, *ArmeoSenso*, *Smart Glove*, and *ArmAssist* are the only commercialized devices on the final list.

## Results

The database search yielded 354 results. After reviewing titles and abstracts and removing duplicates, the results were narrowed down to 110 publications. In full-text review, 8 were excluded for describing assistive devices, 5 were excluded for describing in-clinic devices, 17 were excluded for involving devices that were designed before 2010 or unmodified camera or video game systems, 4 were excluded for describing incomplete or unrealized designs, 5 were excluded for only using virtual reality headsets, and 28 were excluded for being off-topic, such as describing a non-robotic rehabilitation therapy program. Upon reviewing the references of the included publications, 16 additional publications were added by the authors that did not appear in the search but were relevant to the review, bringing the total number of publications to 59.

**Fig. 4:**
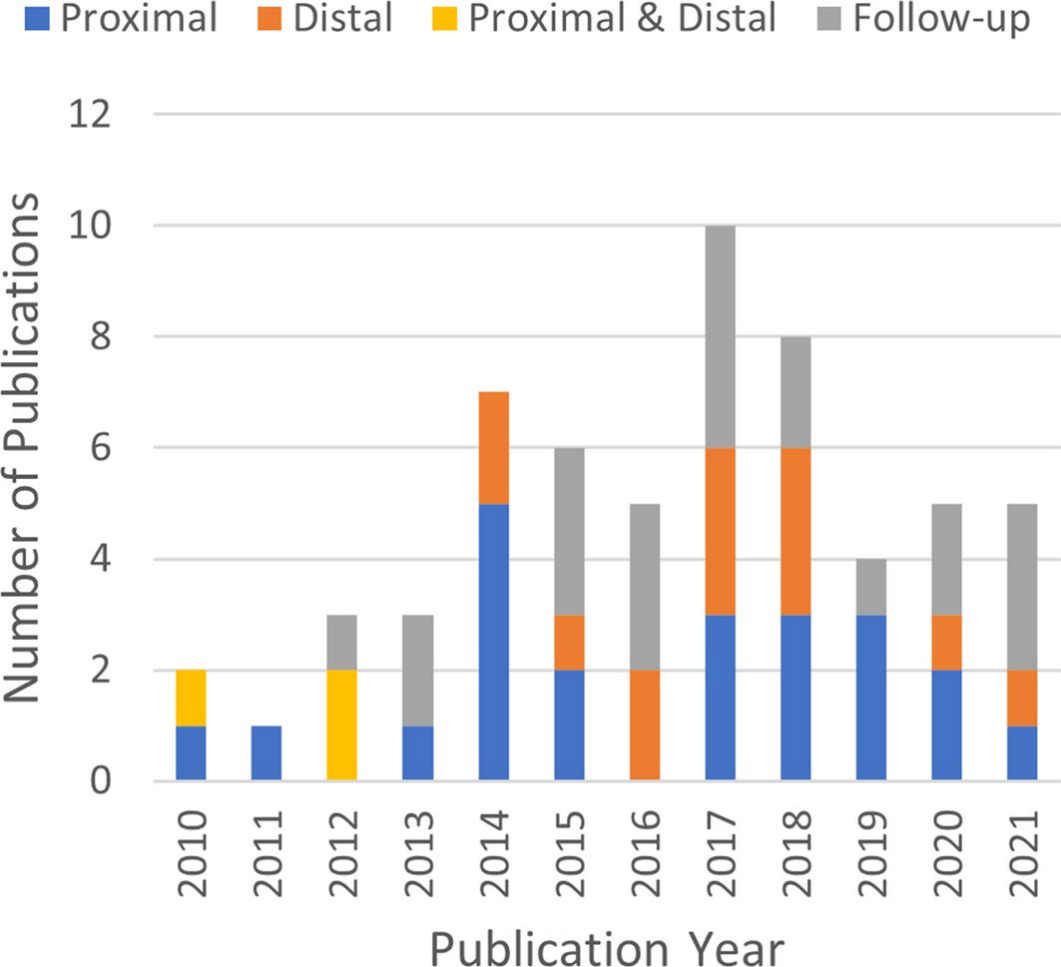
A histogram showing the frequency of publications on home-based rehabilitation mechatronic devices since 2010, colour-coded to distinguish between papers introducing novel devices (and the anatomy targeted by the device), and papers continuing the development of an earlier device. Devices targeting proximal anatomy (shoulder and elbow) are consistently represented across the time span, while devices targeting distal anatomy (wrist and hand) have increased in frequency in the past 6 years

Figure 4 shows a histogram of the included publications. The devices are grouped in the histogram and elsewhere in this work by the anatomy they target. The targeted anatomy is divided into two categories: distal, referring to the wrist and hand, and proximal, referring to the shoulder, elbow, and forearm. Some devices involved both proximal and distal elements and appear in their own separate group. This histogram illustrates the increasing trend in home-based rehabilitation over the time period: 68% more papers were published in the second half of the time period than the first half, and approximately 38% more novel devices were developed. The 38 devices identified are presented in Table 1. This Table is also available as an additional file (Additional file 1).

**Table 1:** Mechatronic devices for home-based upper limb rehabilitation therapy

Orig. year	Device or author	DOF (A/U)	Motions	Actuators	Measurement capability	Type
Proximal						
2010	Ren et al. [42]	1/0	Elbow motion	DC Motor	Elbow joint pos.	Joint-based
2011	Lu et al. [43, 44]	2/0	Horizontal planar reaching	DC Motor	Hand pos.	Endpoint
2013	*RAE* [45]	0/1	Circular reaching	None	Hand pos.	Endpoint
2014	*CR2* [46]	1/0	Linear reaching	DC motor	Hand pos.	Endpoint
2014	*Haptic Theradrive* [47]	1/0	Circular reaching	DC motor	Hand pos. and force	Endpoint
2014	*hCAAR* [37, 48]	2/0	Horizontal planar reaching	Electric motors	Hand pos. and force	Endpoint
2014	*ATD* [49]	3/1	3D reaching and grasping	DC motor + damper	Hand pos.	Endpoint
2014	Tommasino et al. [50]	0/2	Horizontal planar reaching	None	Hand pos. and force	Endpoint
2015	Mohamaddan et al. [51]	2/0	Vertical planar reaching	DC motors	Hand pos.	Endpoint
2015	*ArmeoSenso* [52, 53]	0/5	3D reaching	None	Arm accel. and orient., joint pos., trunk pos.	Joint-based
2017	Wojewoda et al. [54]	0/2	Horizontal planar reaching	None	Hand pos. and orient.	Endpoint
2017	*HOTAR* [55, 56]	2/0	Horizontal planar reaching	DC motors	Hand pos. and force	Endpoint
2017	*BULReD* [57]	2/0	Horizontal planar reaching (bimanual)	DC motors	Hand pos. and force	Endpoint
2018	*PVSED* [58, 59]	1/0	Elbow motion	DC motor + spring	Elbow joint pos.	Joint-based
2018	*HomeRehab* [60, 61]	3/0	3D reaching	DC motors	Hand pos. and force	Endpoint
2018	Gao et al. [62]	3/0	3D reaching	DC motors	Elbow, forearm, and wrist flexion joint pos.	Joint-based
2019	*PaRRo* [63]	0/2	Horizontal planar reaching	Magnetic brakes	Hand pos.	Endpoint
2019	Bai et al. [64]	2/0	Spherical reaching	DC motors	Hand pos., force and torque	Endpoint
2019	*HomeRehabMaster* [65]	0/5	3D reaching	None	Arm accel. and orient., joint pos., trunk pos.	Joint-based
2020	Phan et al. [66]	0/1	Elbow motion	Linear servomotors + spring	Elbow joint pos.	Joint-based
2020	Nicholson-Smith et al. [67]	2/0	Horizontal planar reaching	DC motors	Hand pos. and force	Endpoint
2021	Zhang et al. [68]	6/0	3D reaching (bimanual)	Linear servomotors	Shoulder and elbow joint pos.	Joint-based
Distal						
2014	*SCRIPT* [69–72]	0/6	Wrist flexion, grasping (individual fingers)	None (springs)	Finger and wrist pos., hand pos.	Joint-based
2014	*Physiotherabot/WF* [73, 74]	3/0	Wrist orient.	DC motors	Wrist pos. and hand force	Endpoint
2015	Polygerinos et al. [75]	5/0	Grasping (individual fingers)	Pneumatic bladders	Fingertip forces	Joint-based
2016	Yang et al. [76]	1/0	Grasping	Stepper motor	Fingertip forces, finger flexion	Joint-based
2016	*Smart Glove* [77–80]	0/8	Grasping (individual fingers), 3D reaching	None	Hand position, orientation, and acceleration, wrist position, finger positions	Joint-based
2017	*HandSOME* [81]	0/1	Grasping	None	Grasp pos.	Joint-based
2017	*eWrist* [82, 83]	1/1	Wrist orient.	DC Motor	Wrist orient., velocity, torque, (sEMG)	Joint-based
2017	*QikRehab* [84]	0/6	Object manipulation (pinch, grasp, twist, roll)	None	Hand accel., grip force	Endpoint
2018	*DIAGNOBOT* [85]	3^a^/1	Wrist orient. and grasping	Electric servomotors	Wrist orient., force, torque	Endpoint
2018	*Ambidexter* [86]	3/0	Wrist orient. and grasping	DC motors	Hand orient., grasp force	Endpoint
2018	*X-Glove* [87]	5/3	3D reaching and grasping (individual fingers)	Linear servomotors	Finger pos., arm joint pos.	Joint-based
2020	*PWRR* [88]	2/0	Wrist orient.	Pneumatic pistons	Wrist orient.	Endpoint
2021	*RobHand* [89]	5/0	Grasping (individual fingers)	Linear servomotors	Finger pos.	Joint-based
Proximal + Distal						
2010	*GT System* [90]	0/6	3D reaching and grasping	None	Hand pos. and orient., trunk pos.	Endpoint
2012	*ULERD* [91–93]	3/0	Elbow motion and wrist flexion and rotation	DC motors	Elbow and wrist joint pos.	Joint-based
2012	*ArmAssist* [94–99]	0/3	Horizontal planar reaching and grasping	DC motors	Hand pos., wrist orient., vertical and grasp force	Endpoint^b^

For unnamed devices, the first author’s last name is given. *DOF* degrees of freedom, *A/U* actuated and unactuated

^a^ The *DIAGNOBOT* can be reconfigured to support 3 different degrees of freedom, but only one is active at a time

^b^The *ArmAssist* has a wrist module that gives it both endpoint and joint-based characteristics

**Fig. 5:**
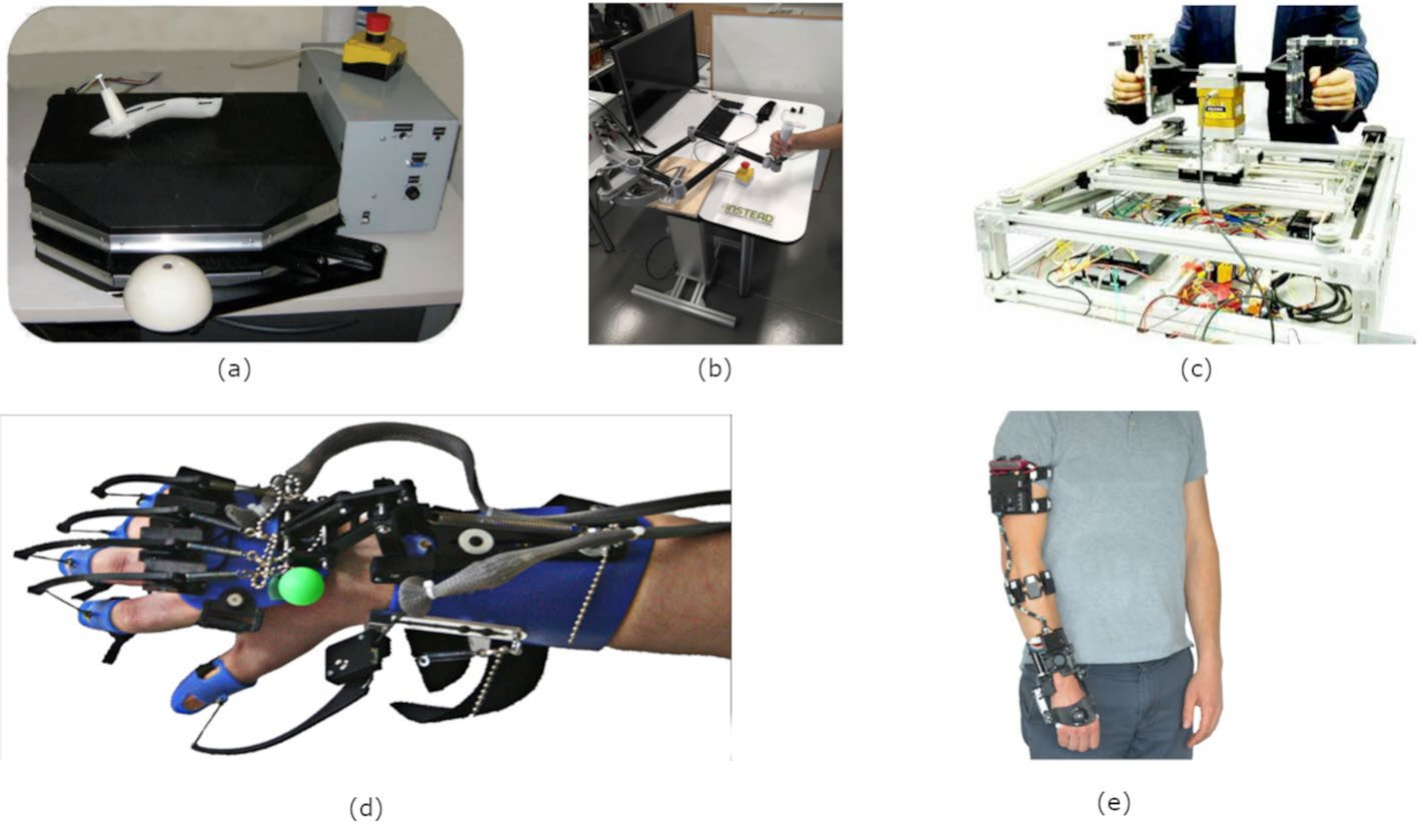
Examples of endpoint mechatronic devices: **a** Lu [44], edited, licensed under CC BY-NC-ND 3.0; **b** *HomeRehab* [60], licensed under CC BY 4.0; **c** *BULReD* [57], licensed under CC BY 4.0. Examples of joint-based devices: **d** *SCRIPT* [72], edited, licensed under CC BY 4.0; **e** *eWrist* [83], edited, licensed under CC BY 4.0

The devices were summarized by their functionality, including the anatomy targeted, the number of degrees of freedom (DOF) offered, the motions allowed, the actuators used in the design (if present), measurement capabilities, and the type of interaction provided. Within each group the devices are arranged by order of their original publication date. The interaction type is divided into two categories: endpoint, referring to devices that interact with the user through a handle or other single point on the body; and joint-based, referring to devices that attach to multiple points across joints on the body. While previous reviews of rehabilitation robots have used the categories “endpoint” and “exoskeleton” [41], we use joint-based as a broader category that includes non-skeletal devices such as wearable sensors. For example, the *ArmeoSenso* attaches sensors at multiple points along the arm, so it does not fit the definition of an endpoint device, yet lacks any skeletal structure to qualify it as an exoskeleton. Therefore, exoskeleton devices are a subset of “joint-based” devices. Examples of devices in each category included in the review are given in Fig. 5. Each motion listed in the table can be thought of as a component of a task. For example, a task to arrange cutlery on a table could be considered as the sum of several reaching and grasping motions.

Quantitative measures of the devices’ characteristics, such as force/torque, workspace dimensions, and cost, are not presented in Table 1 for three reasons. The first is that the devices presented cover a broad range of structural configurations, so that numerical comparisons of their kinematic and dynamic parameters are difficult. Even devices with a similar interaction type and number of DOF, such as the *PaRRo* [63] and the *hCAAR* [48], have different kinematic structures that make a concise, meaningful numerical comparison between them challenging, let alone between more dissimilar devices. The second is that the numerical properties of the devices were not always presented in the publications: the force or torque capabilities of 12 out of the 26 actuated devices were not reported. Only eight devices reported cost estimates of the final design. The third reason is that a qualitative comparison of the devices provides a better view of the functionality of the devices rather than the specific implementation details. The quantitative characteristics of the devices are discussed later in this section based on the limited available information.

### Motions

The following motions were identified for the devices presented in Table 1: grasping, elbow motion, reaching, wrist orientation, and object manipulation. Variations in the motion were distinguished further. Grasping refers to opening and closing the hand, while grasping (individual fingers) indicates that the device is capable of more complicated grasps involving individual fingers. Reaching refers to translation of the hand in space, and is distinguished according to the shape of the workspace the hand can reach. Elbow motion can be considered a simple form of reaching where the device only measures or actuates the elbow joint. Wrist orientation refers to rotations of the wrist and forearm. Object manipulation involves investigatory motions such as pinching, twisting and rolling an object.

Reaching tasks were the most common: 23 out of the 38 devices were designed to perform a reaching task (24 including the devices designed for elbow motion). Of those devices, only six also included grasping, and only two, *BULReD* [57] and the design by Zhang et al. [68], included the unimpaired arm in a bimanual task.

### Degrees of freedom, anatomy, and device type

**Fig. 6:**
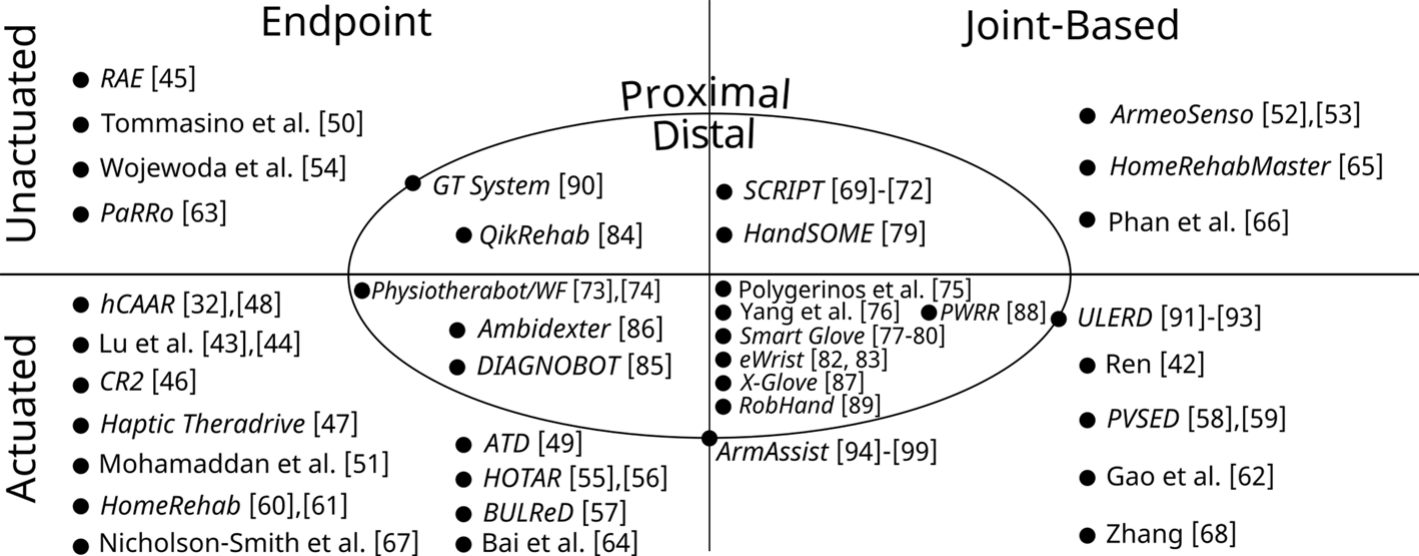
A Venn diagram showing the variety of home-based upper limb rehabilitation devices, grouped according to the anatomy they target (proximal meaning shoulder and elbow, and distal meaning wrist and hand) and the interaction type with the patient (endpoint meaning interaction through a single point, and joint-based meaning interaction through multiple points on the body across joints). Devices that belong to multiple sets are placed on the borders. The device names correspond to the names and references in Table 1

To help visualize the variety of types and anatomy presented in Table 1, the devices are grouped in a Venn diagram in Fig. 6. Each quadrant of the diagram represents a category of devices, for example the upper-left quadrant shows unactuated endpoint-type devices. Devices in the centre target distal anatomy, while devices at the edges target proximal anatomy. Two devices did not fit neatly within the categories of the Venn diagram, namely the *GT System* and *ArmAssist*, therefore they are shown at the borders of the regions.

### Measurement and assessment capabilities

Approximately half of the devices measure the spatial position of the user’s hand, six measure the position of a subset of arm joints, and three measure the position of all arm joints. Three devices, the *ArmeoSenso*, *HomeRehabMaster*, and *GT System*, measure trunk orientation.

Sixteen of the devices measure the force or torque of the user’s interaction. Five of those measure grasping or fingertip force, and nine measure bulk hand force or torque (lifting, pushing, pulling or twisting forces). One device, the *eWrist* [83], measures forearm muscle activation with a surface electromyogram (sEMG) armband.

Out of the 38 devices, 21 proposed a method of assessing patient motor function automatically, 10 of which used multiple forms of assessment. The different methods proposed were: joint range of motion or reachable workspace (11 devices); kinematic performance, such as tracking error, movement time, or maximum jerk (11 devices); required assistance force or patient voluntary force (5 devices); compensation frequency, such as the number of times a patient leans forward excessively (3 devices); game success rate, such as the number of times a patient achieves a desired position or action (3 devices); and virtual reproduction of an existing assessment, specifically a subset of upper limb actions involved in the Fugl-Meyer assessment (1 device, the *HomeRehabMaster* [65]).

### Actuation

Of the 26 actuated devices, all but two used electric motors, and 18 used DC motors specifically. Electric motors, and DC motors in particular, are well-understood from a dynamic modelling perspective, straightforward to control, compact, and can easily be purchased in a variety of sizes, so their popularity in this application is unsurprising. The only non-electric actuated devices found in this review were the soft robotic glove designed by Polygerinos et al. [75] and the *PWRR* [88], both of which used pneumatic actuators.

### Force and torque capability

For the devices that reported force or torque capability, the magnitude varies greatly. Devices targeting reaching motions or proximal anatomy were capable of larger forces and torques than those targeting finer tasks and distal anatomy. For example, the maximum torque of the *ULERD* elbow exoskeleton was 16 N m [93], while the wrist orientation endpoint device *Ambidexter* was capable of 8.4 N m [86].

The strongest devices were the *Haptic Theradrive* [47] and the *BULReD* [57], both of which reported a maximum force of 200 N. That is an order of magnitude larger than the other reaching devices, such as the *ATD* (10–16 N) [49], the *hCAAR* (28 N) [60], and the *PaRRo* (30 N) [63]. The force capability of the *HOTAR* was not specified directly, but in experiments it did not exceed a force of 15 N [56]. Since the *BULReD* was designed for bimanual reaching, it is reasonable that its force capabilities are larger: involving the unimpaired arm in the task increases the force capability of the patient. The *Haptic Theradrive* is an outlier compared to the other unimanual reaching devices, explained by the authors as a budget-related issue: they obtained an aftermarket treadmill motor for a low price that exceeded their requirements [47].

### Cost

Although 24 out of the 38 devices presented mentioned that ‘low cost’ was an important design consideration, only 8 provided a cost estimate. The lowest cost device was that developed by Mohammadan et al., which cost approximately 1000 Malaysian Ringgit [51] (between 200–250 USD). The next most inexpensive was the *GT System* at approximately 1000 USD [90]. The *Haptic Theradrive* [47], *Ambidexter* [86], *PaRRo* [63], and *RobHand* [89] had costs in the 3000–4000 USD range, while the *hCAAR* [48] cost 8400 USD. Following the expected trend, devices with more DOF, and more actuated DOF specifically, tended to be more expensive. While all of the presented costs are lower than similar in-clinic devices, none of the publications provided a metric for determining whether their costs are sufficiently low except for the *RobHand* [89], whose designers consulted with therapists on the feasible device cost.

## Discussion

### Task capabilities

Real-life tasks, such as those in ADL, involve bimanual activity more often than unimanual activity [100], and involve hand activity as well. Bilateral arm training has been found to significantly reduce upper limb motor impairment compared to conventional therapy, particularly for stroke survivors in the chronic phase of stroke, although it does not significantly improve measured function [101]. Only two of the devices presented explicitly involve bimanual activity. Other devices could be involved in a bimanual task, but lack the sensory capabilities to track the second hand or limb. The most likely reason for this limitation is that adding further DOF, such as grasping, increases the cost and complexity of the device. Therefore, the benefit of enabling more complex tasks must be weighed against the cost of making the device more expensive, difficult to operate, and larger in size.

This is where designing the devices with a task or rehabilitation assessment in mind becomes advantageous for designers. By comparing the device’s capabilities against a desired set of tasks, designers and researchers can better justify the design tradeoffs. Linking designs back to the tasks they can perform also helps researchers make more objective comparisons between different designs. Additionally, creating modular designs that can target specific needs of individuals without being one-size-fits-all can be based around specific therapy tasks or activities. Of the devices mentioned, four were designed around specific therapy tasks (reaching), and one, the *HomeRehabMaster* [65], was designed around the Fugl-Meyer Assessment. By considering the necessary sensors, whether built into the device or by using cameras, future devices can be designed for remote assessment and supervision.

This is one area in which mixed actuated and unactuated designs can be used. Almost all of the devices presented were either entirely actuated or entirely unactuated, therefore there is a gap where selectively actuated devices can be developed. Generally speaking, stroke survivors demonstrate obvious impaired motor function on one side of the body, so they primarily need assistance forces on that side. Therefore, designers can expand the capabilities of a device by mixing an actuated design on the patient’s affected side with an unactuated design on the unaffected side. This would allow bimanual therapy, or other activities with more degrees of freedom, without increasing the device cost as much as a fully actuated system. However, more research is needed on the effectiveness of bilateral arm training to determine if this capability is worth the tradeoffs for at-home rehabilitation.

### Safety

Safety is important for any device that a stroke survivor is expected to use unsupervised or remotely supervised. The choice of actuator has significant implications on the safety, performance, and weight of the devices, and therefore represents a challenge in the design. Rigidly connecting the user to a motor creates the potential for injury, especially for stroke survivors with weakened joints and muscles and reduced proprioception. Most of the devices presented use rigid structures to transmit force, with the following exceptions. The *PVSED* [58] introduces a compliant coupling between the user and the actuator. That coupling can then be adjusted to change the stiffness according to the needs of the user. The elbow support device by Phan et al. [66] uses linear actuators to adjust the stiffness of elastic cords running around the elbow, creating adaptive elbow extension assistance. The soft pneumatic glove developed by Polygerinos et al. [75] uses compliant pneumatic bladders to actuate the fingers. By incorporating compliance in their designs, these three devices achieve a level of inherent safety independent of the control system. The downside is that it can increase the weight and complexity of the overall system. For example, pneumatic systems require compressors and regulators in addition to a power supply. Soft robotics is itself a rapidly developing field [102], so compliant designs may become more common in rehabilitation robots. Safety should be central to the design process for any home-based rehabilitation robot; compliant designs, although they add complexity to the control design, are a low-cost, fail-safe way of achieving that.

Although the safety features discussed in the reviewed literature focused largely on compliant actuation, other complementary avenues for ensuring user safety, such as software-based velocity and force limits, could be implemented as well. Given the importance of safety for at-home rehabilitation devices, future literature should describe all potential safety features in the design, whether in hardware or software.

### Control

Control algorithms for rehabilitation robots have three objectives: maintaining safe operation (stability), providing sensory feedback (haptic sensation), and providing therapeutic intervention (assistance/resistance). A wide variety of controllers have been designed to address each of these objectives. Detailed reviews of control algorithms that can be applied to upper limb rehabilitation have been published [41, 103].

The design of a rehabilitation robot determines the types of controllers that can be applied to its operation. For example, some control methods [104–108] require either measurements or accurate estimates of patient hand force to maintain stability. These algorithms are particularly useful for telerehabilitation, specifically physical telepresence, where the therapist can physically interact with a patient during a remote therapy session. Hand force can be measured directly using force sensors, or estimated from EMG [109–111] or force myogram (FMG) [108] sensors on the arm.

Control algorithms that incorporate therapeutic intervention through assistance or resistance forces can require additional sensors to ensure patients remain engaged in the therapy [112]. EMG can be used to detect intent, allowing more intuitive control by the user that matches their effort [112–114], and to prevent over-reliance on the robotic assistance by the patient, improving engagement by maintaining a consistent challenge [115]. Assistance is particularly important for patients with more severe motor impairments, therefore the selection of the control algorithms, and by extension the sensors included on the robot, must be tied to the intended end-user.

These control algorithms not only determine the forms of assistance available, but also have important implications for safety. Atashzar et al. [105] have noted that remote assistance or facilitation involves an energy exchange from the therapist to the patient that would be lost in traditional teleoperation control approaches. Allowing this energy exchange to occur while maintaining system stability requires control algorithms that rely on sensor data such as interaction forces [105]. Designs whose intended applications include remote patient–therapist interaction must consider the requirements of the underlying control algorithms to ensure safety.

Ten of the devices presented in Table 1 included hand force sensors, and although none of them incorporated EMG or FMG directly in their designs, the *eWrist* [82, 83] included an off-the-shelf surface-EMG sensor band. Adding sensors to these designs increases their cost, therefore further investigation into low-cost force sensors is warranted. To that end, determining particular force ranges and resolutions necessary for different therapeutic tasks would help guide the development of low-cost sensors by providing a baseline performance measure.

### Compensation

Only three of the 38 devices were designed to detect compensatory behaviours, such as leaning forward excessively, during therapy activities. Data on trunk orientation in addition to arm joint positions are important for therapists assessing the performance of a patient using these devices. Understanding *how* a patient performs a task is crucial to determining if they are overly reliant on compensation as well as how their recovery is progressing. Compensation involves the patient moving body parts over which they have more control, such as their trunk or shoulders, to compensate for body parts over which they have less control, such as their elbow or wrist [116]. Compensation need not be prevented if the patient is capable of performing the task without risking further injury [33], but the therapist must first be aware of the compensation to make that decision with the patient. Compensation detection can be accomplished using cameras [117], inertial measurement units (IMUs) on the patient’s body [53, 90], or even pressure sensors built-into the patient’s seat [46, 118].

Identifying compensation behaviour from sensor data is a complex task: therapists can identify it visually by the motion of the patient’s whole body as they perform a set of tasks [117], but there is no concrete rule on what constitutes compensation. Therefore, researchers are turning to machine learning [118] based on large datasets labelled by therapists [117] with a large array of sensors observing the patient’s motion to perform automated compensation detection. Such systems can be used to flag potentially problematic compensation during therapy activities for a therapist to review later. Even so, subtle signs of adverse effects, such as patient discomfort or fatigue, can be difficult for an automated system to capture. Adding more sensors increases device cost, but lacking this data may be an unacceptable compromise.

Linking the design of these devices to therapeutic practice can guide designers on what sensory data must be collected. While traditional therapy measures are difficult to translate to robotic systems, novel measures of impairment [119] have been developed that can serve as a guide for the types of data that need to be collected. The necessary set of sensors depends greatly on what assessments the device is expected to be able to perform, whether traditional methods such as the Fugl-Meyer Assessment [65] or novel assessments based on kinematic data [23]. Designers can use the assessments they choose to determine the minimum number and configuration of sensors.

### Target users

Of the 38 devices included in this review, 17 took an entirely anatomical approach to their design, meaning that their design focused exclusively on anthropometric data. Four devices considered some basic user needs, such as space requirements and difficulty donning and doffing the device. Only six devices included user consultations prior to the design, of which only two (the *ArmAssist* and *hCAAR*) consulted patients specifically. This indicates that many of the devices that are presented as rehabilitation robots have limited connection to specific user needs. Tying the design directly to user needs can improve the efficiency of the designs presented.

The suitability of devices for patients at different levels of function depends on the anatomy targeted by the device, the type of interaction it provides, and whether the device is actuated to provide active assistance. Unactuated devices are suitable for patients who have regained much of their upper limb function. Therefore, the devices across the top of Fig. 6 are better suited to patients with low impairment, while those across the bottom are better for those with more severe impairment.

One pattern that emerges from Fig. 6 is that there are far more endpoint-type devices than joint-type. Endpoint devices have been more thoroughly explored, especially for proximal anatomy. This is unsurprising given that proximal-targeted endpoint devices were among the first explored in the clinic-based context as well. In comparison, the joint-type devices are more evenly distributed between the two anatomical categories. Endpoint devices have several advantages over joint-type: they require fewer actuators and sensors to provide motions in a given workspace, and they are easier and safer to operate. However, endpoint devices do not measure arm joint angles or trunk orientation unless they are supported by an external sensor system, therefore they cannot provide important information on the use of compensatory, potentially dysfunctional movement strategies.

Being unactuated allows a device to achieve a large number of degrees of freedom at the expense of being less suitable for patients with severe impairment. The *GT (Gesture Therapy) System* [90] is unactuated and uses a free-floating handle with a grip sensor combined with a camera system to track hand position. Including the grip sensor in the handle allows the *GT System* to involve the patient’s distal as well as their proximal anatomy in therapy. The *Smart Glove* is an entirely unactuated flexible glove-like structure that measures hand motion through accelerometers and individual finger position through the deflection of its flexible structure [78]. The *ArmAssist* [94, 95] is a wheeled arm splint that allows assisted motion across a tabletop, while the latest version [96] includes an unactuated hand module. These devices demonstrate a key insight: including unactuated degrees of freedom allows these devices to be used in more suitable and complex tasks while keeping cost low. Tellingly, the *ArmAssist*’s design includes a detailed analysis of stakeholder needs justifying the approach taken [94]. The question as to which DOF need and do not need actuation should be motivated by the particular needs of the target group: some stroke survivors may need actuated assistance grasping and less assistance with bulk movement of their arm. Involving users in the design, not only in considering their needs but actively seeking their involvement in the design, can lead to more effective designs [120, 121].

Another issue related to the target users is the ability of a device to adapt to a user’s changing needs over time. Increasing challenge and a variety of exercises have been identified as key factors in maintaining user perseverance in at-home technology-supported therapy [38]. The design of a given device affects how the challenge of a therapy task can be adjusted. For example, actuated devices have much more capability to fine-tune challenge: the amount of resistance or assistance of the system can be increased or decreased in addition to the complexity of the task. In comparison, unactuated devices rely on increasing or decreasing the complexity of the task to increase or decrease the associated challenge. In either case, the amount and approach to changing the difficulty is task-specific and remains an open question.

### Force and torque capability

While a force range of 10–30 N seems to be the consensus for unimanual reaching tasks, the reasons for this force range were not well explained in the literature. The *ATD* explains the design of its passive springs to allow gravity compensation [49], but otherwise the reaching devices did not have clear justifications for this force range. Since the design of most of the devices presented was not motivated by a specific set of tasks or exercises, it is difficult to determine the appropriateness of the forces presented. Force and torque capabilities are an aspect of the design of rehabilitation robots that should be motivated by the nature of the tasks the robot is expected to perform and the expected level of patient impairment so that clear performance baselines can be established and compared.

### Motivation and engagement

Only a few of the devices approached the issue of motivation and engagement: the *hCAAR* [37, 48], *HomeRehabMaster* [65], *SCRIPT* [71], and *ArmAssist* [97] each describe serious games designed for the purpose of motivating their users. Motivation and engagement are particularly important in unsupervised environments, such as the home environment, where patients are responsible for maintaining their own therapy regimens. Motivation and engagement are affected by such factors as [122]: attention, where multisensory feedback informs the user on areas to improve; adaptation, where the difficulty can adapt to the abilities of the user; engagement, where users feel a sense of reward and a connection with their rehabilitation goals; and socialization, where users feel connected to others. Future designs should incorporate these factors.

### Affordability

Cost is a complex metric to consider for the design of at-home rehabilitation robots. Cost is difficult to estimate for an experimental device: the costs presented in the reviewed publications only reflect hardware costs which could be eclipsed by the labour costs in a commercial setting. As a concrete example, the device designed by Lu et al. had a retail price of approximately 10,000 USD, compared to the original project material cost of approximately 3000 USD [123].

Affordability, the cost relative to a person’s ability to pay, should be considered in designs rather than cost alone. The target users for these devices are often dependent on insurance or government assistance to purchase such a device. It is therefore helpful for researchers and developers to compare their designs to locally available support programs, and to include that information as part of their design. Retailers of future rehabilitation robots may be able to lease devices to users, thereby reducing the up-front cost, however this approach still places the cost on the stroke survivors’ ability to pay.

### Future direction

Given the gaps identified in the previous subsections, there is a need for devices allowing more complex motions related to real-world activities, but the limitations of the home-based setting make this difficult. One approach is to combine unactuated and actuated designs to allow more complex tasks while keeping the cost low. Different combinations of actuated and unactuated DOF could help patients with different needs and impairments. Proximal joint-based sensing through IMUs (such as in the *ArmeoSenso*) or cameras (such as in *HomeRehabMaster*) can provide arm and body pose information to supplement the capabilities of actuated endpoint devices, while body-mounted cameras could potentially give joint-based devices hand position measurement capability [124]. To determine which DOF must be un-/actuated, devices should be designed with a specific set of therapy tasks and assessments, as well as the users’ capabilities, in mind. Bimanual tasks could be made more accessible with this approach, using unactuated components for the unaffected limb and actuated components for the affected limb, while not increasing the cost and complexity as much as a fully actuated design. Machine learning can be applied to this wealth of sensor data to detect compensation or evaluate performance, helping therapists track their patients’ progress.

The user needs, both in terms of the specific physical impairments they experience and the tasks they wish to practise, should be central to the design of any device. However, stroke survivors’ recovery trajectory varies, therefore following a ‘one-size-fits-all’ approach is challenging. Devices that can change as the user’s ability changes will have broader applicability and be more attractive. By having the user only learn one device over the course of their recovery, less time is spent training them on the use of different devices. Modular designs that can be easily reconfigured as the patient progresses could meet a more diverse set of user needs.

Furthermore, as researchers develop new designs they should endeavour to relate their work directly to the needs of the relevant stakeholders. As previously discussed, it is difficult to analyse the performance of the different devices presented in the literature because many of those devices are not designed for any specific rehabilitation therapy. Instead, it seems that they are designed to target specific anatomy and future rehabilitative therapies must be designed to fit the hardware. While this approach is reasonable, it may lead to reduced efficiency as the effectiveness of a given design can only be evaluated after an appropriate therapy has been designed for it. By taking the opposite approach, and basing designs on task-based therapies, or functional assessments, that are already known to be effective, researchers can evaluate the effectiveness of their designs earlier in the development process.

Beyond considering the needs of users, the needs of the latest control algorithms need to be considered in the design depending on its application. As mentioned in the Control subsection of the Discussion, devices used for telerehabilitation, where the therapist and the patient can physically interact remotely through a robotic system, require additional sensors and accurate actuation to maintain patient–robot and robot–therapist interaction stability and safety. Such devices also require the means to establish a reliable network connection to allow the exchange of video, audio, force, and position data. In other words, devices primarily intended for independent therapy practice will differ from devices intended for remote-controlled therapy systems. Therefore, researchers should articulate the specific intended application of a given design beyond it being a home-based rehabilitation robot.

### Limitations

The limitations of the review are as follows. The review was conducted in English, and only three databases were searched. The filtering of results was conducted by a single author. The search terms may not have covered all possible combinations or terminology for home-based rehabilitation robots. However, given these limitations we are confident that even if some devices were not discovered in the search, the categories and properties identified would not change.

## Conclusion

Interest in the area of home-based rehabilitation mechatronics has increased in the past 12 years, driven by the maturation of in-clinic rehabilitation mechatronic technology and a growing need to improve therapy access in patients’ homes without increasing the cost. Existing devices largely focus on reaching motions and have an insufficient sensory capability to evaluate critical aspects of task performance, such as detecting compensation. Very few of the publications reviewed described a stakeholder consultation process prior to or during the design. Even fewer mentioned consulting stroke survivors specifically. Clearly linking the design features to identified user needs is important to both ensure that those needs are met and to allow the effectiveness of the designs to to be evaluated. Future designs should more clearly link to the therapy tasks they are capable of, the impairments for which they assist, their measurement capabilities, and their cost, relative to the specific needs of patients and therapists. Future designs should also explore a mix of actuated and unactuated degrees of freedom to increase the capabilities of devices without greatly increasing the cost.

## Supplementary Information

**Additional file 1**. Mechatronic devices for home-based upper limb rehabilitation therapy.

## Data Availability

The datasets generated and/or analysed during the current study are not publicly available but are available from the corresponding author on reasonable request.

## Abbreviations

ADL: Activities of daily life
DOF: Degrees of freedom
EMG: Electromyography or electromyograph or electromyogram
FMG: Force myography or force myograph or force myogram
IMU: Inertial measurement unit
ICF: The World Health Organization’s International Classification of Functioning, Disability, and Health
USD: United States Dollars

## References

[1] Feigin VL, Norrving B, Mensah GA (2017). Global burden of stroke. Circul Res..

[2] Chatterji S, Byles J, Cutler D, Seeman T, Verdes E (2015). Health, functioning, and disability in older adults-present status and future implications. The Lancet..

[3] Public Health Agency of Canada. Stroke in Canada: Highlights from the Canadian Chronic Disease Surveillance [Web Page.]; 2017. Available from: https://www.canada.ca/content/dam/phac-aspc/documents/services/publications/diseases-conditions/stroke-vasculaires/stroke-vasculaires-canada-eng.pdf.

[4] Lawrence ES, Coshall C, Dundas R, Stewart J, Rudd AG, Howard R (2001). Estimates of the prevalence of acute stroke impairments and disability in a multiethnic population. Stroke.

[5] Kwakkel G, Kollen BJ, der Grond JVV, Prevo AJH (2003). Probability of regaining dexterity in the flaccid upper limb: impact of severity of paresis and time since onset in acute stroke. Stroke.

[6] Nichols-Larsen DS, Clark PC, Zeringue A, Greenspan A, Blanton S (2005). Factors influencing stroke survivors’ quality of life during subacute recovery. Stroke.

[7] Dimyan MA, Cohen LG (2011). Neuroplasticity in the context of motor rehabilitation after stroke. Nat Rev Neurol.

[8] Lang CE, MacDonald JR, Gnip C (2007). Counting repetitions: an observational study of outpatient therapy for people with hemiparesis post-stroke. J Neurol Phys Ther.

[9] Kleim JA, Jones TA (2008). Principles of experience-dependent neural plasticity: implications for rehabilitation after brain damage. J Speech Lang Hear Res.

[10] Ruis DA, Polhemus RW, Book WJ. inventors. Robotic exercise machine and method. 1980;4:235–437.

[11] Krebs HI, Volpe BT, Williams D, Celestino J, Charles SK, Lynch D (2007). Robot-aided neurorehabilitation: a robot for wrist rehabilitation. IEEE Trans Neural Syst Rehabil Eng.

[12] Staubli P, Nef T, Klamroth-Marganska V, Riener R. Effects of intensive arm training with the rehabilitation robot ARMin II in chronic stroke patients: Four single-cases. J NeuroEng Rehabil. 2009;6. 10.1186/1743-0003-6-46 PMC2807864 20017939

[13] Book W, Ruis D, Polhemus R. Microprocessor controlled robotic exercise machine for athletics and rehabilitation. In: Proc. of the 1979 Joint Automatic Control Conference; 1979:771–776.

[14] Hogan N, Krebs HI, Charnnarong J, Srikrishna P, Sharon A. MIT-MANUS: a workstation for manual therapy and training. I. In: Proc. of the IEEE International Workshop on Robot and Human Communication; 1992. p. 161–165.

[15] Hogan N, Krebs HI, Sharon A, Jain C (1995). inventors. Interactive robotic therapist..

[16] Nef T, Mihelj M, Riener R (2007). ARMin: a robot for patient-cooperative arm therapy. Med Biol Eng Compu.

[17] Nef T, Guidali M, Riener R (2009). ARMin III—arm therapy exoskeleton with an ergonomic shoulder actuation. Appl Bionics Biomech..

[18] Guidali M, Duschau-Wicke A, Broggi S, Klamroth-Marganska V, Nef T, Riener R (2011). A robotic system to train activities of daily living in a virtual environment. Med Biol Eng Compu.

[19] Aisen ML, Krebs HI, Hogan N, McDowell F, Volpe BT (1997). The effect of robot-assisted therapy and rehabilitative training on motor recovery following stroke. Arch Neurol.

[20] Mehrholz J, Pohl M, Platz T, Kugler J, Elsner B. Electromechanical and robot-assisted arm training for improving activities of daily living, arm function, and arm muscle strength after stroke. Cochrane Database Syst Rev. 2018. 10.1002/14651858.CD006876.pub5 PMC6513114 30175845

[21] Chen Z, Wang C, Fan W, Gu M, Yasin G, Xiao S (2020). Robot-assisted arm training versus therapist-mediated training after stroke: a systematic review and meta-analysis. J Healthc Eng..

[22] Rodgers H, Bosomworth H, Krebs HI, van Wijck F, Howel D, Wilson N (2019). Robot assisted training for the upper limb after stroke (RATULS): a multicentre randomised controlled trial. The Lancet..

[23] Mostafavi SM, Scott S, Dukelow S, Mousavi P (2017). Reduction of assessment time for stroke-related impairments using robotic evaluation. IEEE Trans Neural Syst Rehabil Eng.

[24] Zhang M, Zhang S, McDaid A, Davies C, Xie SQ (2018). Automated objective robot-assisted assessment of wrist passive ranges of motion. J Biomech.

[25] Chen Y, Poole MC, Olesovsky SV, Champagne AA, Harrison KA, Nashed JY (2021). Robotic assessment of upper limb function in a nonhuman primate model of chronic stroke. Transl Stroke Res.

[26] Winstein CJ, Stein J, Arena R, Bates B, Cherney LR, Cramer SC (2016). Guidelines for adult stroke rehabilitation and recovery. Stroke.

[27] Maciejasz P, Eschweiler J, Gerlach-Hahn K, Jansen-Troy A, Leonhardt S (2014). A survey on robotic devices for upper limb rehabilitation. J Neuroeng Rehabil.

[28] Chen Y, Abel KT, Janecek JT, Chen Y, Zheng K, Cramer SC (2019). Home-based technologies for stroke rehabilitation: a systematic review. Int J Med Informatics.

[29] Hall RE, French E, Khan F, Zhou L, Linkewich B, Willems D, et al. Ontario stroke evaluation report 2016: a focus on stroke rehabilitation. Institute for Clinical Evaluative Sciences; 2016.

[30] Moreland JD, Depaul VG, Dehueck AL, Pagliuso SA, Yip DWC, Pollock BJ (2009). Needs assessment of individuals with stroke after discharge from hospital stratified by acute Functional Independence Measure score. Disabil Rehabil.

[31] Langstaff C, Martin C, Brown G, McGuinness D, Mather J, Loshaw J (2015). Enhancing community-based rehabilitation for stroke survivors: creating a discharge link. Top Stroke Rehabil.

[32] Public Health Agency of Canada. Vulnerable populations and COVID-19 [Web Page]; 2020. Available from: https://www.canada.ca/en/public-health/services/publications/diseases-conditions/vulnerable-populations-covid-19.html.

[33] Hebert D, Lindsay MP, McIntyre A, Kirton A, Rumney PG, Bagg S (2016). Canadian stroke best practice recommendations: stroke rehabilitation practice guidelines, update 2015. Int J Stroke.

[34] Nilsen DM, Gillen G, Geller D, Hreha K, Osei E, Saleem GT (2014). Effectiveness of interventions to improve occupational performance of people with motor impairments after stroke: an evidence-based review. Am J Occup Ther.

[35] Radomski MV, Anheluk M, Arulanantham C, Finkelstein M, Flinn N (2018). Implementing evidence-based practice: A context analysis to examine use of task-based approaches to upper-limb rehabilitation. Br J Occup Ther.

[36] Sivan M, Gallagher J, Holt R, Weightman A, Levesley M, Bhakta B (2014). Investigating the international classification of functioning, disability, and health (ICF) framework to capture user needs in the concept stage of rehabilitation technology development. Assist Technol.

[37] Sivan M, Gallagher J, Holt R, Weightman A, O’Connor R, Levesley M (2016). Employing the International Classification of Functioning, Disability and Health framework to capture user feedback in the design and testing stage of development of home-based arm rehabilitation technology. Assist Technol.

[38] Neibling BA, Jackson SM, Hayward KS, Barker RN (2021). Perseverance with technology-facilitated home-based upper limb practice after stroke: a systematic mixed studies review. J Neuroeng Rehabil.

[39] Towards a Common Language for Functioning, Disability and Health. World Health Organization; 2002. Available from: https://www.who.int/publications/m/item/icf-beginner-s-guide-towards-a-common-language-for-functioning-disability-and-health.

[40] Cameron ID (2010). Models of rehabilitation—commonalities of interventions that work and of those that do not. Disabil Rehabil.

[41] Atashzar SF, Shahbazi M, Patel RV (2019). Haptics-enabled Interactive NeuroRehabilitation Mechatronics: classification, functionality, challenges and ongoing research. Mechatronics.

[42] Ren Y, Park HS, Li Y, Wang L, Zhang LQ. A wearable robot for upper limb rehabilitation of patients with neurological disorders. In: Proc. of the IEEE International Conference on Robotics and Biomimetics; 2010. p. 64–68.

[43] Lu EC, Wang RH, Hebert D, Boger J, Galea MP, Mihailidis A (2011). The development of an upper limb stroke rehabilitation robot: identification of clinical practices and design requirements through a survey of therapists. Disabil Rehabil Assist Technol.

[44] Lu EC, Wang R, Huq R, Gardner D, Karam P, Zabjek K (2012). Development of a robotic device for upper limb stroke rehabilitation: a user-centered design approach. Paladyn J Behav Robot..

[45] Zondervan DK, Palafox L, Hernandez J, Reinkensmeyer DJ (2013). The resonating arm exerciser: design and pilot testing of a mechanically passive rehabilitation device that mimics robotic active assistance. J Neuroeng Rehabil.

[46] Khor KX, Rahman HA, Fu SK, Sim LS, Yeong CF, Su ELM (2014). A novel hybrid rehabilitation robot for upper and lower limbs rehabilitation training. Proc Comput Sci..

[47] Theriault A, Nagurka M, Johnson MJ (2014). Design and development of an affordable haptic robot with force-feedback and compliant actuation to improve therapy for patients with severe hemiparesis. IEEE Trans Haptics.

[48] Sivan M, Gallagher J, Makower S, Keeling D, Bhakta B, O’Connor RJ (2014). Home-based Computer Assisted Arm Rehabilitation (hCAAR) robotic device for upper limb exercise after stroke: results of a feasibility study in home setting. J Neuroeng Rehabil.

[49] Westerveld AJ, Aalderink BJ, Hagedoorn W, Buijze M, Schouten AC, Kooij HVD (2014). A damper driven robotic end-point manipulator for functional rehabilitation exercises after stroke. IEEE Trans Biomed Eng.

[50] Tommasino P, Melendez-Calderon A, Burdet E, Campolo D (2014). Motor adaptation with passive machines: a first study on the effect of real and virtual stiffness. Comput Methods Programs Biomed.

[51] Mohamaddan S, Jamali A, Abidin ASZ, Jamaludin MS, Majid NAA, Ashari MF (2015). Development of upper limb rehabilitation robot device for home setting. Proc Comput Sci..

[52] Wittmann F, Lambercy O, Gonzenbach RR, Raai MAV, Hover R, Held J, et al. Assessment-driven arm therapy at home using an IMU-based virtual reality system. In: Proc. of the IEEE International Conference on Rehabilitation Robotics; 2015. p. 707–712.

[53] Wittmann F, Held JP, Lambercy O, Starkey ML, Curt A, Höver R (2016). Self-directed arm therapy at home after stroke with a sensor-based virtual reality training system. J Neuroeng Rehabil.

[54] Wojewoda KK, Culmer PR, Gallagher JF, Jackson AE, Levesley MC. Hybrid position and orientation tracking for a passive rehabilitation table-top robot. In: Proc. of the IEEE International Conference on Rehabilitation Robotics; 2017. p. 702–707. 10.1109/ICORR.2017.8009330 28813902

[55] Ogata K, Hirabayashi Y, Kubota K, Tsuji T. Home rehabilitation assist robot to facilitate isolated movements for hemiplegia patients. In: Proc. of the IEEE International Conference on Intelligent Robots and Systems; 2017. p. 527–532.

[56] Ogata K, Hirabayashi Y, Kubota K, Hasegawa Y, Tsuji T. Rehabilitation for hemiplegia using an upper limb training system based on a force direction. In: Proc. of the IEEE International Conference on Rehabilitation Robotics; 2017. p. 533–538. 10.1109/ICORR.2017.8009303 28813875

[57] Miao Q, Zhang M, Wang Y, Xie SQ. Design and interaction control of a new bilateral upper-limb rehabilitation device. J Healthc Eng. 2017; 1–9. 10.1155/2017/7640325 PMC5632482 29104747

[58] Liu Y, Guo S, Hirata H, Ishihara H, Tamiya T (2018). Development of a powered variable-stiffness exoskeleton device for elbow rehabilitation. Biomed Microdevice.

[59] Liu Y, Guo S, Yang Z. Performance evaluation of a powered variable-stiffness exoskeleton device for bilateral training. In: Proc. of the IEEE International Conference on Mechatronics and Automation; 2019. p. 2163–2167.

[60] Díaz I, Catalan JM, Badesa FJ, Justo X, Lledo LD, Ugartemendia A (2018). Development of a robotic device for post-stroke home tele-rehabilitation. Adv Mech Eng.

[61] Catalan JM, Garcia JV, Lopez D, Diez J, Blanco A, Lledo LD, et al. Patient evaluation of an upper-limb rehabilitation robotic device for home use. In: Proc. of the IEEE International Conference on Biomedical Robotics and Biomechatronics; 2018. p. 450–455.

[62] Gao B, Wei C, Guo S, Xiao N, Bu D, Xu H, et al. Embedded system-based a portable upper limb rehabilitation robot. In: Proc. of the IEEE International Conference on Mechatronics and Automation; 2018. p. 631–636.

[63] Washabaugh EP, Guo J, kang Chang C, Remy CD, Krishnan C (2019). A portable passive rehabilitation robot for upper-extremity functional resistance training. IEEE Trans Biomed Eng.

[64] Bai J, Song A, Wang T, Li H (2019). A novel backstepping adaptive impedance control for an upper limb rehabilitation robot. Comput Electr Eng.

[65] Bai J, Song A (2019). Development of a novel home based multi-scene upper limb rehabilitation training and evaluation system for post-stroke patients. IEEE Access..

[66] Phan TQ, Nguyen H, Mulyk A, Vermillion BC, Lee SW. Development of self-adaptable mechanism to compensate angle-dependent flexor tone of the elbow joint post-stroke: a pilot study. In: Proc. of the International Conference of the IEEE Engineering in Medicine & Biology Society (EMBC); 2020. p. 4779–4782. 10.1109/EMBC44109.2020.9176501 33019059

[67] Nicholson-Smith C, Mehrabi V, Atashzar SF, Patel RV (2020). A multi-functional lower- and upper-limb stroke rehabilitation robot. IEEE Trans Med Robot Bionics..

[68] Zhang S, Huang M, Wang L, Liao KL, Yang R. Design of an auxiliary device for home-based stroke rehabilitation. In: Proc. of the IEEE Global Conference on Life Sciences and Technologies (LifeTech); 2021. p. 135–138.

[69] Amirabdollahian F, Ates S, Basteris A, Cesario A, Buurke J, Hermens H (2014). Design, development and deployment of a hand/wrist exoskeleton for home-based rehabilitation after stroke— SCRIPT project. Robotica.

[70] Ates S, Mora-Moreno I, Wessels M, Stienen AHA. Combined active wrist and hand orthosis for home use: Lessons learned. Proc of the IEEE International Conference on Rehabilitation Robotics. 2015;p. 398–403.

[71] Nijenhuis SM, Prange GB, Amirabdollahian F, Sale P, Infarinato F, Nasr N, et al. Feasibility study into self-administered training at home using an arm and hand device with motivational gaming environment in chronic stroke. J NeuroEng Rehabil. 2015;12. 10.1186/s12984-015-0080-y PMC4599772 26452749

[72] Ates S, Haarman CJW, Stienen AHA (2017). SCRIPT passive orthosis: design of interactive hand and wrist exoskeleton for rehabilitation at home after stroke. Auton Robot.

[73] Atlihan M, Akdogan E, Arslan MS. Development of a therapeutic exercise robot for wrist and forearm rehabilitation. In: Proc. of the International Conference on Methods and Models in Automation and Robotics (MMAR); 2014. p. 52–57.

[74] Akdoğan E, Aktan ME, Koru AT, Arslan MS, Atlıhan M, Kuran B (2018). Hybrid impedance control of a robot manipulator for wrist and forearm rehabilitation: performance analysis and clinical results. Mechatronics.

[75] Polygerinos P, Wang Z, Galloway KC, Wood RJ, Walsh CJ (2015). Soft robotic glove for combined assistance and at-home rehabilitation. Robot Auton Syst.

[76] Yang J, Xie H, Shi J (2016). A novel motion-coupling design for a jointless tendon-driven finger exoskeleton for rehabilitation. Mech Mach Theory.

[77] Shin JH, Kim MY, Lee JY, Jeon YJ, Kim S, Lee S (2016). Effects of virtual reality-based rehabilitation on distal upper extremity function and health-related quality of life: a single-blinded, randomized controlled trial. J Neuroeng Rehabil.

[78] Jung HT, Kim H, Jeong J, Jeon B, Ryu T, Kim Y. Feasibility of using the RAPAEL Smart Glove in upper limb physical therapy for patients after stroke: A randomized controlled trial. IEEE; 2017. p. 3856–3859. 10.1109/EMBC.2017.8037698 29060739

[79] Lee SH, Lee JY, Kim MY, Jeon YJ, Kim S, Shin JH (2018). Virtual reality rehabilitation with functional electrical stimulation improves upper extremity function in patients with chronic stroke: a pilot randomized controlled study. Arch Phys Med Rehabil.

[80] Kang MG, Yun SJ, Lee SY, Oh BM, Lee HH, Lee SU (2020). Effects of upper-extremity rehabilitation using smart glove in patients with subacute stroke: results of a prematurely terminated multicenter randomized controlled trial. Front Neurol.

[81] Chen J, Nichols D, Brokaw EB, Lum PS (2017). Home-based therapy after stroke using the hand spring operated movement enhancer (HandSOME). IEEE Trans Neural Syst Rehabil Eng.

[82] Lambelet C, Lyu M, Woolley D, Gassert R, Wenderoth N. The eWrist—a wearable wrist exoskeleton with sEMG-based force control for stroke rehabilitation. Proc of the IEEE International Conference on Rehabilitation Robotics. 2017; 726–733. 10.1109/ICORR.2017.8009334 28813906

[83] Lambelet C, Temiraliuly D, Siegenthaler M, Wirth M, Woolley DG, Lambercy O (2020). Characterization and wearability evaluation of a fully portable wrist exoskeleton for unsupervised training after stroke. J Neuroeng Rehabil.

[84] Jie S, Haoyong Y, Chaw TL, Chiang CC, Vijayavenkataraman S. An interactive upper limb rehab device for elderly stroke patients. In: Proc. of the 27th CIRP Design Conference. 2017;60:488–493.

[85] Aktan ME, Akdoğan E (2018). Design and control of a diagnosis and treatment aimed robotic platform for wrist and forearm rehabilitation: DIAGNOBOT. Adv Mech Eng.

[86] Wai CC, Leong TC, Gujral M, Hung J, Hui TS, Wen KK. Ambidexter: A low cost portable home-based robotic rehabilitation device for training fine motor skills. In: Proc. of the IEEE International Conference on Biomedical Robotics and Biomechatronics; 2018. p. 420–425.

[87] Ghassemi M, Ochoa JM, Yuan N, Tsoupikova D, Kamper D. Development of an integrated actuated hand orthosis and virtual reality system for home-based rehabilitation. In: Proc. of the International Conference of the IEEE Engineering in Medicine and Biology Society; 2018. p. 1689–1692. 10.1109/EMBC.2018.8512704 30440720

[88] Zhang L, Li J, Cui Y, Dong M, Fang B, Zhang P (2020). Design and performance analysis of a parallel wrist rehabilitation robot (PWRR). Robot Auton Syst.

[89] Moreno-SanJuan V, Cisnal A, Fraile JC, Pérez-Turiel J, la Fuente ED (2021). Design and characterization of a lightweight underactuated RACA hand exoskeleton for neurorehabilitation. Robot Auton Syst.

[90] Sucar LE, Luis R, Leder R, Hernández J, Sánchez I. Gesture therapy: a vision-based system for upper extremity stroke rehabilitation. In: Proc. of the International Conference of the IEEE Engineering in Medicine and Biology Society; 2010. p. 3690–3693. 10.1109/IEMBS.2010.5627458 21096856

[91] Song Z, Guo S (2012). Design process of exoskeleton rehabilitation device and implementation of bilateral upper limb motor movement. J Med Biol Eng.

[92] Wei W, Guo S, Zhang W, Guo J, Wang Y. A novel VR-based upper limb rehabilitation robot system. In: Proc. of the ICME International Conference on Complex Medical Engineering. vol. 5. IEEE; 2013. p. 302–306.

[93] Zhang S, Guo S, Gao B, Hirata H, Ishihara H (2015). Design of a novel telerehabilitation system with a force-sensing mechanism. Sensors..

[94] Perry JC, Zabaleta H, Belloso A, Rodriguez-De-Pablo C, Cavallaro FI, Keller T. ArmAssist: Development of a functional prototype for at-home telerehabilitation of post-stroke arm impairment. In: Proc. of the IEEE International Conference on Biomedical Robotics and Biomechatronics; 2012. p. 1561–1566.

[95] Jung JH, Valencia DB, Rodriguez-De-Pablo C, Keller T, Perry JC. Development of a powered mobile module for the ArmAssist home-based telerehabilitation platform. In: Proc. of the IEEE International Conference on Rehabilitation Robotics; 2013. p. 1–6. 10.1109/ICORR.2013.6650424 24187242

[96] Perry JC, Trimble S, Machado LGC, Schroeder JS, Belloso A, Rodriguez-De-Pablo C, et al. Design of a spring-assisted exoskeleton module for wrist and hand rehabilitation. In: Proc. of the International Conference of the IEEE Engineering in Medicine and Biology Society; 2016. p. 594–597. 10.1109/EMBC.2016.7590772 28268400

[97] Rozevink SG, van der Sluis CK, Garzo A, Keller T, Hijmans JM (2021). HoMEcare aRm rehabiLItatioN (MERLIN): telerehabilitation using an unactuated device based on serious games improves the upper limb function in chronic stroke. J Neuroeng Rehabil.

[98] Rozevink SG, van der Sluis CK, Hijmans JM (2021). HoMEcare aRm rehabiLItatioN (MERLIN): preliminary evidence of long term effects of telerehabilitation using an unactuated training device on upper limb function after stroke. J Neuroeng Rehabil.

[99] Guillén-Climent S, Garzo A, Muñoz-Alcaraz MN, Casado-Adam P, Arcas-Ruiz-Ruano J, Mejías-Ruiz M (2021). A usability study in patients with stroke using MERLIN, a robotic system based on serious games for upper limb rehabilitation in the home setting. J Neuroeng Rehabil.

[100] Kilbreath SL, Heard RC (2005). Frequency of hand use in healthy older persons. Aust J Physiothera..

[101] Chen S, Qiu Y, Bassile CC, Lee A, Chen R. Xu D. Effectiveness and success factors of bilateral arm training after stroke: a systematic review and meta-analysis. Front Aging Neurosci. 2022. p. 14. 10.3389/fnagi.2022.875794 PMC9082277 35547621

[102] Polygerinos P, Correll N, Morin SA, Mosadegh B, Onal CD, Petersen K, et al. Soft Robotics: Review of fluid-driven intrinsically soft devices; manufacturing, sensing, control, and applications in human-robot interaction. Adv Eng Mater. 2017;19.

[103] Proietti T, Crocher V, Roby-Brami A, Jarrasse N (2016). Upper-limb robotic exoskeletons for neurorehabilitation: a review on control strategies. IEEE Rev Biomed Eng.

[104] Tadele TS, Vries TJAD, Stramigioli S. Combining energy and power based safety metrics in controller design for domestic robots. Proc of the IEEE International Conference on Robotics and Automation. 2014;p. 1209–1214.

[105] Atashzar SF, Shahbazi M, Tavakoli M, Patel RV (2017). A passivity-based approach for stable patient-robot interaction in haptics-enabled rehabilitation systems: modulated Time-Domain Passivity Control. IEEE Trans Control Syst Technol.

[106] Raiola G, Cardenas CA, Tadele TS, Vries TD, Stramigioli S (2018). Development of a safety- and energy-aware impedance controller for collaborative robots. IEEE Robot Automation Lett.

[107] Ramos A, Hashtrudi-Zaad K. Estimation of upper-limb energy absorption capabilities for stable human-robot interactions. IEEE Haptics Symposium: HAPTICS; 2020. p. 115–20.

[108] Ramos A, Hashtrudi-Zaad K. Estimation of energy absorption capability of arm using force myography for stable human-machine interaction. Proc of the International Conference of the IEEE Engineering in Medicine and Biology Society, EMBS. 2020;p. 4758–4761. 10.1109/EMBC44109.2020.9175410 33019054

[109] Hashemi J, Morin E, Mousavi P, Mountjoy K, Hashtrudi-Zaad K (2012). EMG-force modeling using parallel cascade identification. J Electromyogr Kinesiol.

[110] Johns G, Morin E, Hashtrudi-Zaad K (2016). Force modelling of upper limb biomechanics using ensemble fast orthogonal search on high-density electromyography. IEEE Trans Neural Syst Rehabil Eng.

[111] Johns G, Morin E, Hashtrudi-Zaad K. The role of electromechanical delay in modelling the EMG-force relationship during quasi-dynamic contractions of the upper-limb. Proc of the International Conference of the IEEE Engineering in Medicine and Biology Society. 2016;p. 3634–3637. 10.1109/EMBC.2016.7591515 28269082

[112] Pehlivan AU, Losey DP, Rose CG, O’Malley MK. Maintaining subject engagement during robotic rehabilitation with a minimal assist-as-needed (mAAN) controller. IEEE International Conference on Rehabilitation Robotics. 2017;p. 62–67. 10.1109/ICORR.2017.8009222 28813794

[113] Pehlivan AU, Losey DP, Omalley MK (2016). Minimal assist-as-needed controller for upper limb robotic rehabilitation. IEEE Trans Rob.

[114] Losey DP, McDonald CG, Battaglia E, O’Malley MK (2018). A review of intent detection, arbitration, and communication aspects of shared control for physical human-robot interaction. Appl Mech Rev.

[115] Marchal-Crespo L, Reinkensmeyer DJ. Review of control strategies for robotic movement training after neurologic injury. J NeuroEng Rehabil. 2009;6. 10.1186/1743-0003-6-20 PMC2710333 19531254

[116] Levin MF, Kleim JA, Wolf SL (2009). What do motor “Recovery” and “Compensation” mean in patients following stroke?. Neurorehabil Neural Repair.

[117] Foreman MH, Engsberg JR (2019). A virtual reality tool for measuring and shaping trunk compensation for persons with stroke: design and initial feasibility testing. J Rehabil Assist Technol Eng.

[118] Cai S, Li G, Zhang X, Huang S, Zheng H, Ma K (2019). Detecting compensatory movements of stroke survivors using pressure distribution data and machine learning algorithms. J Neuroeng Rehabil.

[119] Tyryshkin K, Coderre AM, Glasgow JI, Herter TM, Bagg SD, Dukelow SP (2014). A robotic object hitting task to quantify sensorimotor impairments in participants with stroke. J Neuroeng Rehabil.

[120] Harrison SL, Brooks D (2016). Active patient engagement: long overdue in rehabilitation research. Physiother Can.

[121] Ienca M, Kressig RW, Jotterand F, Elger B (2017). Proactive Ethical Design for neuroengineering, assistive and rehabilitation technologies: the Cybathlon Lesson. J Neuroeng Rehabil.

[122] Amorim P, Santos BS, Dias P, Silva S, Martins H (2020). Serious games for stroke telerehabilitation of upper limb—a review for future research. Int J Telerehabil.

[123] Lu EC. Development of an upper limb robotic device for stroke rehabilitation [Ph.D. Dissertation]. University of Toronto; 2011.

[124] Visee RJ, Likitlersuang J, Zariffa J (2020). An effective and efficient method for detecting hands in egocentric videos for rehabilitation applications. IEEE Trans Neural Syst Rehabil Eng.

